# Changes in carbohydrate metabolism and endogenous hormone regulation during bulblet initiation and development in *Lycoris radiata*

**DOI:** 10.1186/s12870-020-02394-4

**Published:** 2020-04-25

**Authors:** Junxu Xu, Qingzhu Li, Liuyan Yang, Xin Li, Zhen Wang, Yongchun Zhang

**Affiliations:** Forestry and Pomology Research Institute, Shanghai Academy of Agricultural Sciences, Shanghai, 201403 China

**Keywords:** *Lycoris radiata*, Bulblet, Propagation efficiency, Carbohydrate, Hormone

## Abstract

**Background:**

*Lycoris* species have great ornamental and medicinal values; however, their low regeneration efficiency seriously restricts their commercial production. Understanding the mechanism of bulblet propagation in this genus, which has remained underexplored to date, could provide a theoretical basis for improving the reproductive efficiency. Therefore, we studied the bulblet initiation and developmental processes in *Lycoris radiata*.

**Results:**

We found that bulblets are formed on the junctions of the innermost layers of scales and the basal plate, and initially present as an axillary bud and gradually develop into a bulblet. We also determined the changes in carbohydrate and endogenous hormone contents during bulblet initiation and development, as well as the expression patterns of genes involved in carbohydrate metabolism and hormone biosynthesis and signaling through transcriptome analysis. Soluble sugars derived from starch degradation in the outer scales are transported to and promote bulblet initiation and development through starch synthesis in the inner scales. This process is mediated by several genes involved in carbohydrate metabolism, especially genes encoding ADP glucose pyrophosphorylase, a crucial starch synthesis enzyme. As for hormones, endogenous IAA, GA, and ABA content showed an increase and decrease during bulblet initiation and development, respectively, which were consistent with the expression patterns of genes involved in IAA, GA, and ABA synthesis and signal transduction. In addition, a decrease in ZR content may be down- and up-regulated by CK biosynthesis and degradation related genes, respectively, with increasing auxin content. Furthermore, expression levels of genes related to BR, JA, and SA biosynthesis were increased, while that of ethylene biosynthesis genes was decreased, which was also consistent with the expression patterns of their signal transduction genes.

**Conclusions:**

The present study provides insights into the effect of carbohydrate metabolism and endogenous hormone regulation on control of *L. radiata* bulblet initiation and development. Based on the results, we propose several suggestions to improve *L. radiata* propagation efficiency in production, which will provide directions for future research.

## Background

The genus *Lycoris* comprises approximately 20 species, which are distributed in the warm temperate and subtropical zones of East Asia [1] and mainly in southwestern China and Japan. *Lycoris* species have high ornamental value and display an exceptionally wide diversity of flower colors [2]. They also have high medicinal value, and alkaloids isolated from their bulbs inhibit viruses, inflammation, tumors, and cancers [3, 4]. Thus, this genus has great potential for commercial development. However, their regeneration efficiency is low. In recent years, wild *Lycoris* resources in China have been overexploited to satisfy the increasing demand for *Lycoris* bulbs [5]. Thus, improving the reproductive efficiency of *Lycoris* species, which may be mainly determined by bulblet propagation efficiency, is extremely important. In *Lycoris* species, bulblets are formed from the axils of the scales, and then gradually develop into a bulb [6]. The ability to produce bulblets differs among species; it is the strongest in *Lycoris radiata*, followed by *Lycoris sprengeri* and *Lycoris aurea*. Bulblet differentiation is regulated by several factors in the study of other flowering bulbs, including carbohydrate metabolism and endogenous hormone regulation [7–9], which has remained underexplored in *Lycoris* to date.

Carbohydrate metabolism is crucial for bulblet formation and development. In flowering bulbs, the bulb is filled with several compounds, a major one of which is starch, which serves as a carbon sink. The starch is degraded into soluble sugars that provide carbon and energy for plant morphogenesis, e.g., the emergence and development of leaves and flower buds [7]. In *Lycoris* species, the starch granules available for degradation in the mother bulb sections serve as an energy source for bulblet initiation and development [6]. During scale cutting propagation of *Lilium*, the starch content in the mother scales declines, while in the bulblets, it increases simultaneously [10]. Studies of the changes in carbohydrate compounds during bulblet development in *Lilium* revealed strong regulation of sucrose and starch metabolism during this process [7, 11, 12]. However, our knowledge of the role of carbohydrate metabolism in *Lycoris* during bulblet formation and development is very limited.

The bulblet regeneration process in *Lycoris* species is very similar to the axillary bud initiation process in species such as rice, Arabidopsis, petunia, and pea [6]. In these species, bud outgrowth is regulated by the interaction of environmental and endogenous signals, such as plant hormones [13]. Auxin and strigolactones (SLs) inhibit bud outgrowth, whereas cytokinin (CK) promotes bud outgrowth [14, 15]. Hormone signaling in bud outgrowth is mainly integrated by the transcription factors TEOSINTE BRANCHED1 (TB1)/BRANCHED1 (BRC1): SLs promote, whereas CK inhibits their expression [16]. In addition, a role for abscisic acid (ABA) has recently come into focus as it was demonstrated that BRC1 promotes ABA accumulation through transcriptional activation of homeobox protein (HB)21, HB40, HB53, and 9-cis-epoxycarotenoid dioxygenase (NCED)3, thus inhibiting bud development [17, 18]. Furthermore, gibberellic acid (GA) and brassinosteroids (BRs) may respectively inhibit and promote bud outgrowth [19]. However, reports on hormone regulation during *Lycoris* bulblet regeneration are rather limited. Recent research on *Lilium* bulblet growth suggested that increasing auxin while lowering cytokinin contents might be useful to promote bulblet growth and development, whereas increasing cytokinin alone can be used to promote bulblet initiation [20, 21]. Additionally, recent studies have revealed that GA promotes shoot growth and multiplication, whereas ABA, daminozide, and chlorocholine chloride significantly improved bulblet quality parameters, such as average size [22, 23]. In addition, in *Lilium*, exogenously applied paclobutrazol, an inhibitor of GA biosynthesis, had an inhibitory effect that was commensurate with the concentration on the aerial and root parts of the bulblets, with low concentrations promoting and high concentrations inhibiting bulblet development [23]. However, these studies evaluated the effects of exogenously applied hormones on bulblet development; changes in endogenous hormone regulation during bulblet formation in *Lycoris* remain to be analyzed.

In the present work, we studied the bulblet initiation and development processes in *Lycoris radiata*, and we investigated changes in carbohydrate content, starch synthesis, metabolic enzyme activity, endogenous hormone contents, and the expression patterns of genes related to carbohydrate metabolism, hormone synthesis, and signal transduction, during these processes. We expected our results to provide a better understanding of the regulation of carbohydrates and hormones during bulblet initiation and development in *L. radiata*, which would be useful for future research on improving the reproductive efficiency of *Lycoris* species.

## Results

### Morphological description of bulblet initiation and development in *Lycoris radiata*

**Fig. 1:**
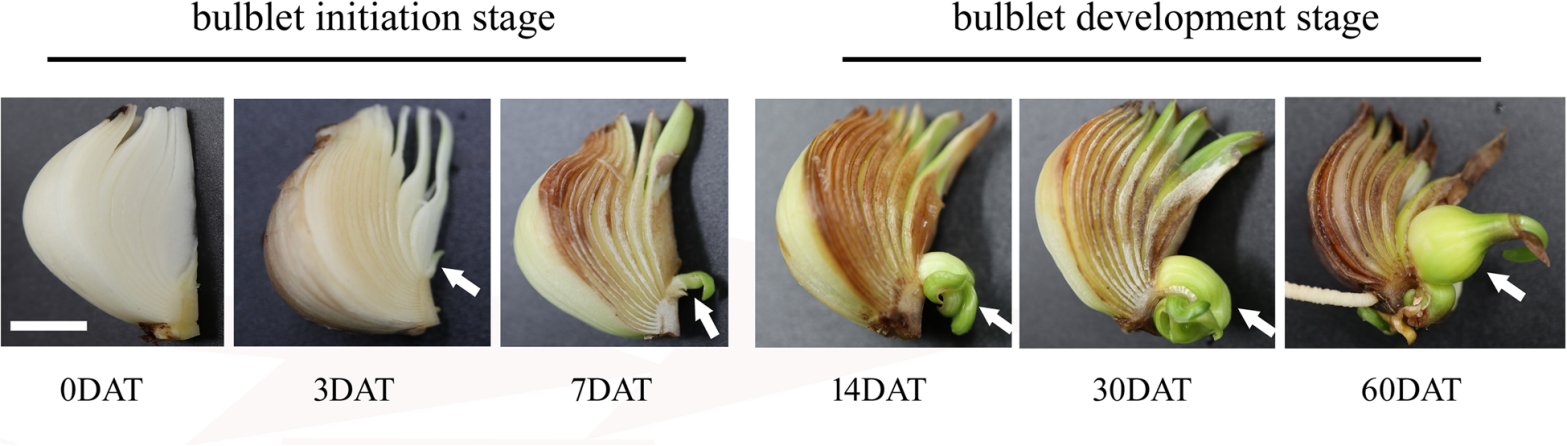
Morphological analysis of initiation and development of a bulblet in a bulb section of *L. radiata*. Arrows indicate the position where the axillary bud is formed and the bulblet develops. Pictures were taken on 0, 3, 7, 14, 30, and 60 DAT. Bar = 1 cm

Based on a two-month observation of bulblet formation in sections prepared from *L. radiata* bulbs collected from the experimental base at the Shanghai Academy of Agricultural Sciences, the process of bulblet formation can be divided into two stages: bulblet initiation (0–7 days after treatment (DAT)) and bulblet development (7–60 DAT). Bulblets form from axillary buds, which are formed on junctions of the innermost layer of scales and the basal plate at 3 DAT (Fig. 1). The newly formed axillary bud elongates and gradually develops into a bulblet from 7 DAT (Fig. 1).

### Transcriptome sequencing, *de-novo* assembly, and functional annotation of unigenes

For RNA-sequencing (RNA-seq) analysis of *L. radiata* bulblet initiation and development, the zones where axillary buds form or newly formed bulblets were sampled on 0, 1, 3, 7, 14, and 30 DAT (termed B0, B1, B3, B7, B14, and B30, respectively), including three biological replicates. RNA-seq yielded 70–77 million raw reads, with more than 6.7 Gb of data for each library (Additional file 1: Table S1). After the elimination of low-quality reads and adaptor sequences, 66–71 million clean reads per library were obtained and used for *de-novo* assembly, with the Q20 and Q30 percentages being higher than 97 and 89%, respectively (Additional file 1: Table S1). All contigs were assembled into 114,638 non-redundant unigenes with an average length of 1406 bp (N50 = 1946 bp) (Additional file 2: Table S2).

All the unigenes were annotated based on BLASTx against seven public databases, including Nr, Nt, SwissProt, KEGG, KOG, Pfam, and GO. In total, 94,264 unigenes (82.23% of the total genes) were annotated in at least one database, and the number of annotated unigenes in each database is shown in Table 1. Among these, 12,732 unigenes (11.11% of the total genes) were annotated in all databases.

**Table 1:** Annotation of unigenes in seven public databases

Annotation database	Number of annotated unigenes	Percentage (%)
Total	114,638	100%
NR	91,636	79.94%
NT	71,222	62.13%
Swissprot	71,466	62.34%
KEGG	74,644	65.11%
KOG	74,754	65.21%
Pfam	70,109	61.16%
GO	23,342	20.36%
Intersection	12,732	11.11%
Overall	94,264	82.23%

### Analysis of differentially expressed genes (DEGs)

**Fig. 2:**
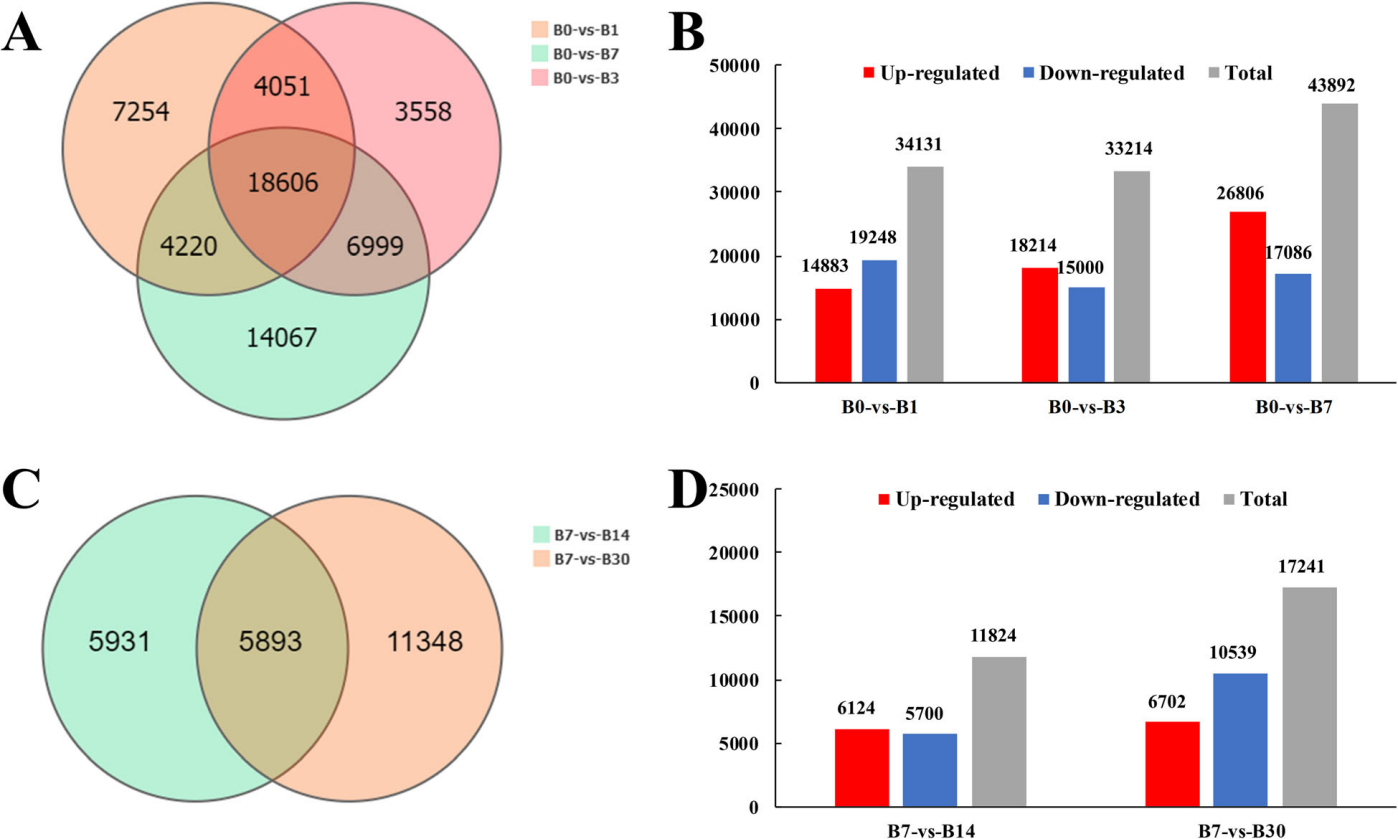
Overview of the numbers of DEGs during bulblet initiation and bulblet development. **a**, **b**: Comparisons of B0 vs. B1, B0 vs. B3, and B0 vs. B7. **c**, **d**: Comparisons of B7 vs. B14 and B14 vs. B30. **a**, **c**: Venn diagrams of shared and unique DEGs in the indicated groups. **b**, **d**: Histograms showing the numbers of up- and downregulated DEGs in the indicated groups. The red columns indicate up-regulated DEGs, and the blue columns represent down-regulated DEGs

We identified DEGs during the bulblet initiation (B0, B1, B3, and B7) and developmental (B14 and B30) stages. DEGs in the initiation stage were compared among sub-sample groups: B0 vs. B1, B0 vs. B3, and B0 vs. B7. In total, 34,131, 33,214, and 43,892 genes were differentially expressed in B0 vs. B1, B0 vs. B3, and B0 vs. B7, respectively (Fig. 2a, b). In addition, 7254, 3558, and 14,067 DEGs were specific for B0 vs. B1, B0 vs. B3, and B0 vs. B7, respectively, and 18,606 DEGs were identified in all three comparisons (Fig. 2a). DEGs in the developmental stage were also compared among sub-sample groups, i.e., B7 vs. B14 and B14 vs. B30, to more aptly reflect changes in gene expression during this stage. In total, 11,824 and 17,241 DEGs were identified for B7 vs. B14 and B14 vs. B30, respectively, which was significantly less than the number of DEGs identified during the bulblet initiation stage (Fig. 2c, d).

### Changes in starch and soluble sugar contents in bulblets and mother scales during bulblet initiation and development in *L. radiata*

**Fig. 3:**
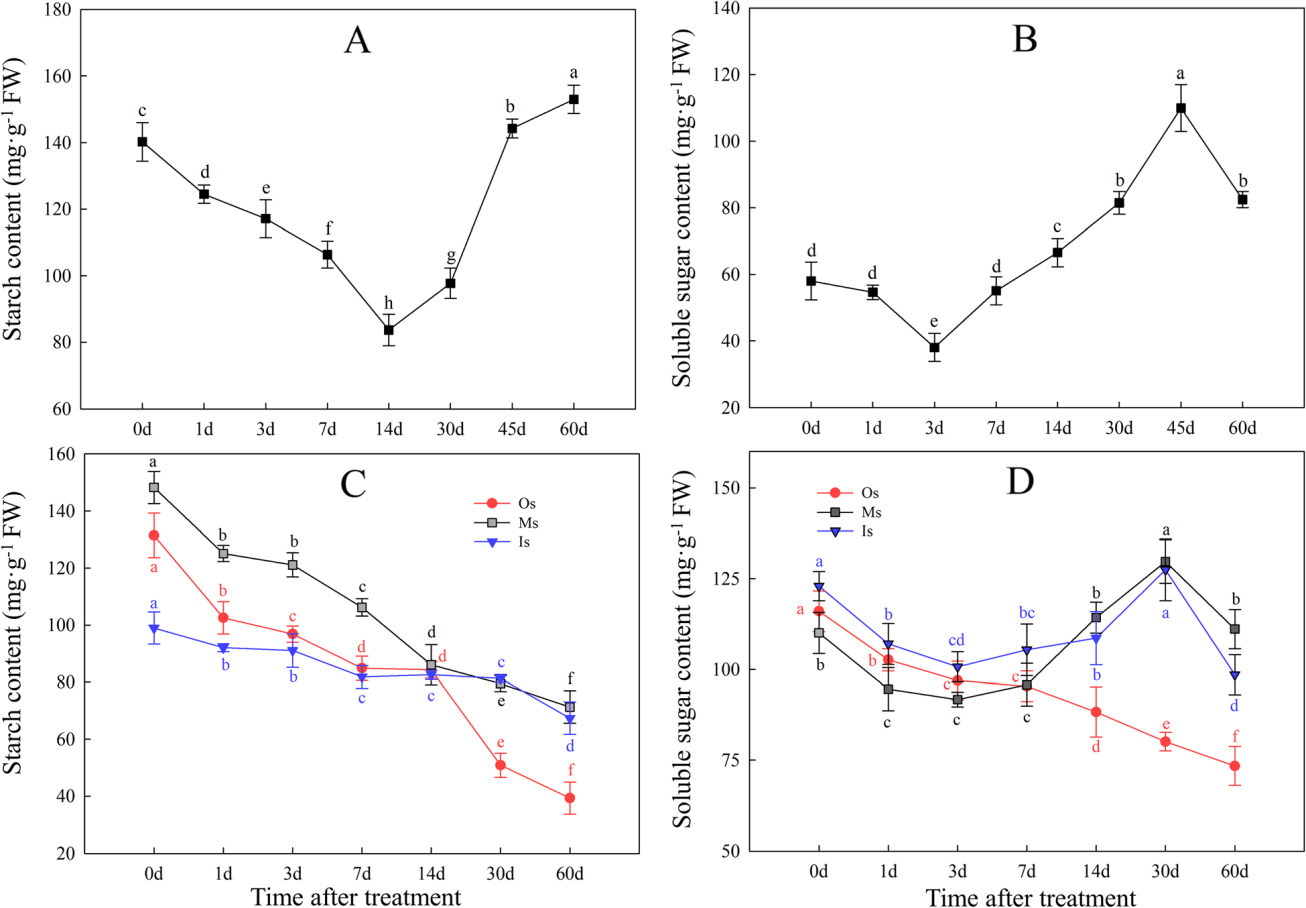
Changes in starch (**a**, **c**) and soluble sugars (**b**, **d**) contents during bulblet initiation and development. **a**, **b**: bulblets and zones where bulblets are formed; **c**, **d**: outer, middle, and inner layers of mother scales. Os: outer scales; Ms.: middle scales; Is: inner scales. Data are shown as the mean ± standard error (n = 3), different letters represent significant differences of the means (*P* < 0.05)

During bulblet initiation and development, starch contents first decreased, reaching a nadir at 14 DAT, and then increased (Fig. 3a). Soluble sugar contents decreased over the first 3 DAT and then increased, peaking at 45 DAT (Fig. 3b).

A previous study revealed that mother bulb sections may serve as an energy source for bulblet initiation and development [6], and we previously reported that mother scales can be divided into three layers, each of which may differently contribute to bulblet initiation and development [24]. Therefore, we also determined changes in carbohydrate contents in the outer, middle, and inner layers of mother scales during bulblet initiation and development (Fig. 3c, d). We found that starch contents in the middle and, especially, the outer scales continuously strongly decreased (Fig. 3c), whereas the starch content in the inner scales decreased at a lower rate than that in the other layers (Fig. 3c). Changes in soluble sugar contents were similar in the middle and inner scales, showing a slight decrease and a subsequent increase (Fig. 3d); however, the sugar content in the outer scales continuously decreased, which was similar to the changes in starch content (Fig. 3d).

### DEGs related to carbohydrate metabolism

**Fig. 4:**
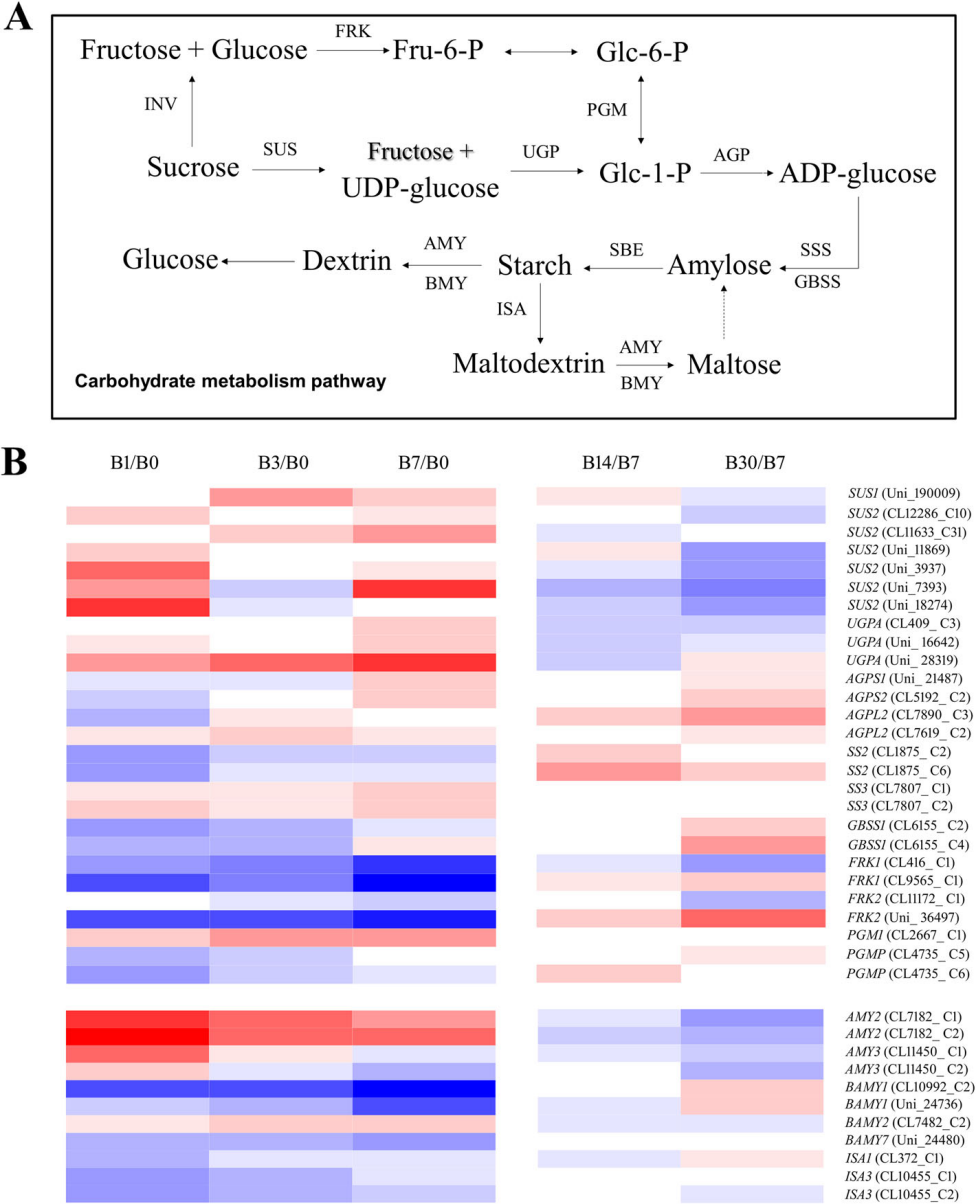
Carbohydrate metabolism during bulblet initiation and development. **a**: simplified overview of carbohydrate metabolism. **b**: Heatmap of the expression of DEGs related to carbohydrate metabolism in the indicated groups during bulblet initiation and development, respectively. The color scale corresponds to log_2_-transformed (fragments per kb per million reads) FPKM values, with red indicating upregulation and blue indicating downregulation. Each row represents a unigene. B0–B30 represent different cDNA libraries obtained from samples collected at different days after treatment. Abbreviations: SUS, sucrose synthase; UGP, UTP-glucose-1-phosphate uridylyltransferase; AGP, glucose-1-phosphate adenylyltransferase; SSS, soluble starch synthase; GBSS, granule-bound starch synthase; SBE, starch branching enzyme; INV, invertase; FRK, fructokinase; PGM, phosphoglucomutase; AMY, alpha-amylase; BMY, beta amylase; ISA, isoamylase.

We identified 38 genes related to carbohydrate metabolism that were differentially expressed in at least one comparison (Additional file 3: Table S3), and their expression patterns are shown in the heatmap in Fig. 4b. Among these DEGs, seven genes encoding sucrose synthase (*SUS*) and three genes encoding UDP-glucose pyrophosphorylase (UGPase) were significantly upregulated during bulblet initiation, but downregulated during bulblet development, and this trend was especially obvious for *SUS2* (Uni_7393) and UGPA (Uni_28319) (Fig. 4b). In addition, except for *SS3* (CL7807_C1 and C2) and *AGPL2* (CL7619_C2), several genes encoding starch synthesis enzymes, including *SS2* (CL1875_C2 and C6), *GBSS1* (CL6155_C2 and C4), *AGPS2* (CL5192_C2), and *AGPL2* (CL7890_C3), encoding starch synthase (SS), granule-bound starch synthase (GBSS), and ADP glucose pyrophosphorylase (AGPase) small and large subunits, respectively, showed expression patterns opposite to those of the *SUS* genes; these genes were mainly downregulated or their expression did not significantly change during bulblet initiation, whereas they were upregulated during bulblet development.

Sucrose can also be metabolized via another pathway that is mediated by invertase (INV), fructokinase (FRK), and phosphoglucomutase (PGM) (Fig. 4a). However, during bulblet initiation, genes encoding these enzymes, including *FRK1–2* and *PGMP*, were significantly downregulated (Fig. 4b), implying that the SUS and UGPase pathway may be the major sucrose metabolic pathway during bulblet initiation in *L. radiata*.

Starch can be degraded into glucose and maltose by alpha-amylase (AMY), beta amylase (BMY), and isoamylase (ISA) (Fig. 4a), leading to a reduction in starch content. In our study, nearly all genes encoding starch metabolism enzymes, especially *BAMY1* (CL10992_C2) and *ISA3* (CL10455_C2), were significantly downregulated during bulblet initiation (Fig. 4b).

### Starch synthesis enzyme activities and gene expression during bulblet formation

**Fig. 5:**
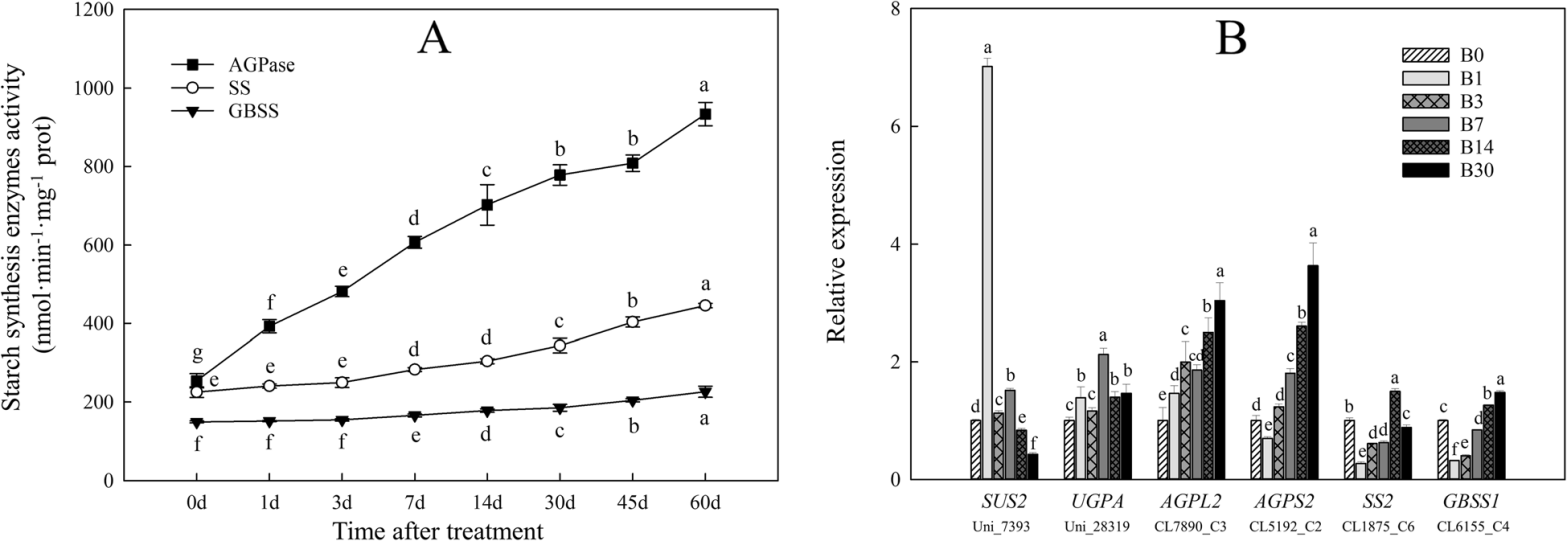
Changes in the activities of three starch synthesis enzymes (**a**) and the expression levels of six starch synthesis-related genes (**b**) during bulblet initiation and development. B0–B30 represent the cDNA libraries obtained from samples collected at the indicated DATs. For RT-qPCR analysis, the values obtained for the B0 samples were arbitrarily set at 1.0. Data are the mean ± standard error (*n* = 3), different letters indicate significant differences of the means (*P* < 0.05)

We measured changes in the activity of three starch synthesis enzymes (AGPase, SS, and GBSS) during bulblet initiation and development. AGPase activity showed the most significant increase among the three enzymes throughout the developmental process (Fig. 5a). SS and GBSS activities showed no significant changes during bulblet initiation, but increased during bulblet development (Fig. 5a).

We selected six candidate genes for verification of the RNA-seq data by quantitative reverse transcription (qRT)-PCR, the results of which were in good agreement with the RNA-seq results (Fig. 5b). *SUS2* and *UGPA* showed significant increases in expression during bulblet initiation, followed by a decrease during bulblet development (Fig. 5b). Interestingly, the expression patterns of *SS2* and *GBSS1* were opposite to those of *SUS2* and *UGPA* (Fig. 5b). Different from the other four genes, expression levels of *AGPL2* and *AGPS2* genes, which encode the AGPase large and small subunits, increased throughout the bulblet initiation and developmental stages (Fig. 5b), which was consistent with the changes in AGPase activity.

### Changes in the contents of four endogenous hormones during bulblet formation

**Fig. 6:**
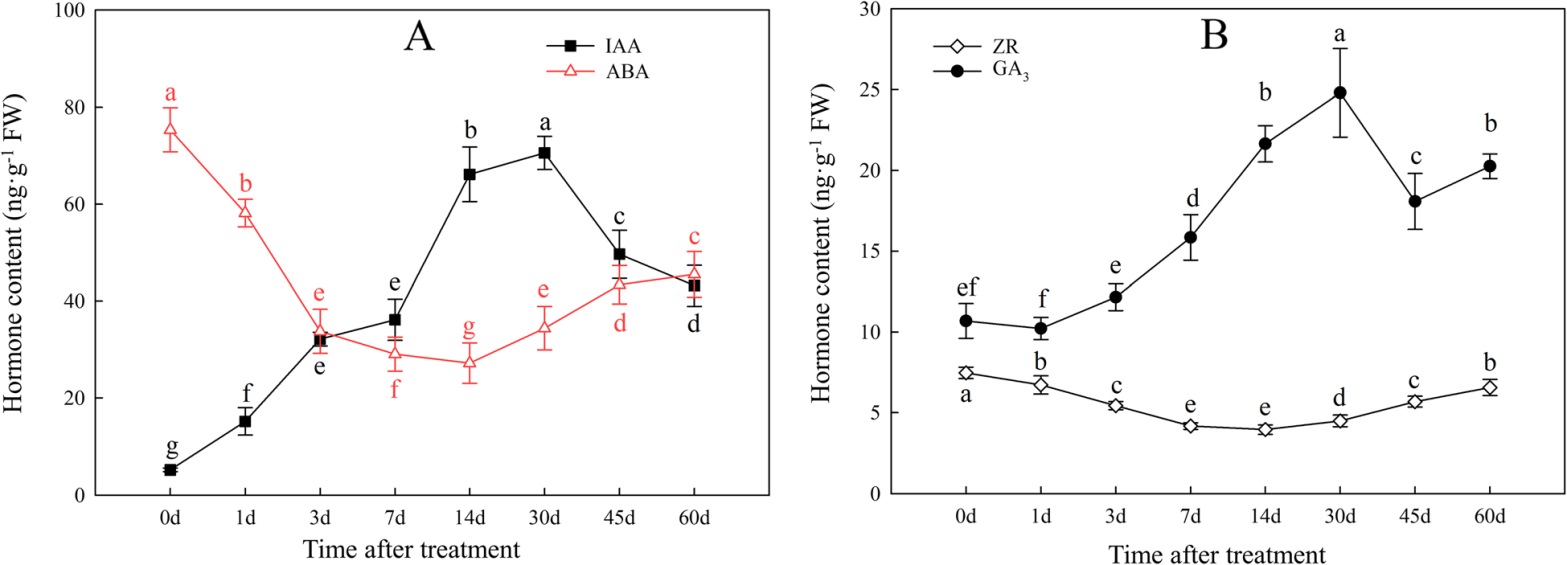
Changes in endogenous hormone contents during bulblet initiation and development. **a**: IAA and ABA contents; **b**: ZR and GA_3_ contents. Data are the mean ± standard error (*n* = 3), different letters indicate significant differences of the means (*P* < 0.05)

We measured changes in the contents of four major endogenous hormones, including indole-3-acetic acid (IAA; the most common auxin), zeatin riboside (ZR; a type of cytokinin), GA_3_, and ABA, during bulblet initiation and development. The IAA and GA_3_ contents increased from 0 to 30 DAT and then decreased, whereas the GA_3_ content increased at a lower rate than IAA over the first 3 DAT (Fig. 6a, b). Interestingly, changes in the ZR and ABA contents were quite similar; these hormones first showed a quick decrease from 0 to 14 DAT, and then slightly increased (Fig. 6a, b).

### DEGs related to hormone biosynthesis and signal transduction

**Fig. 7:**
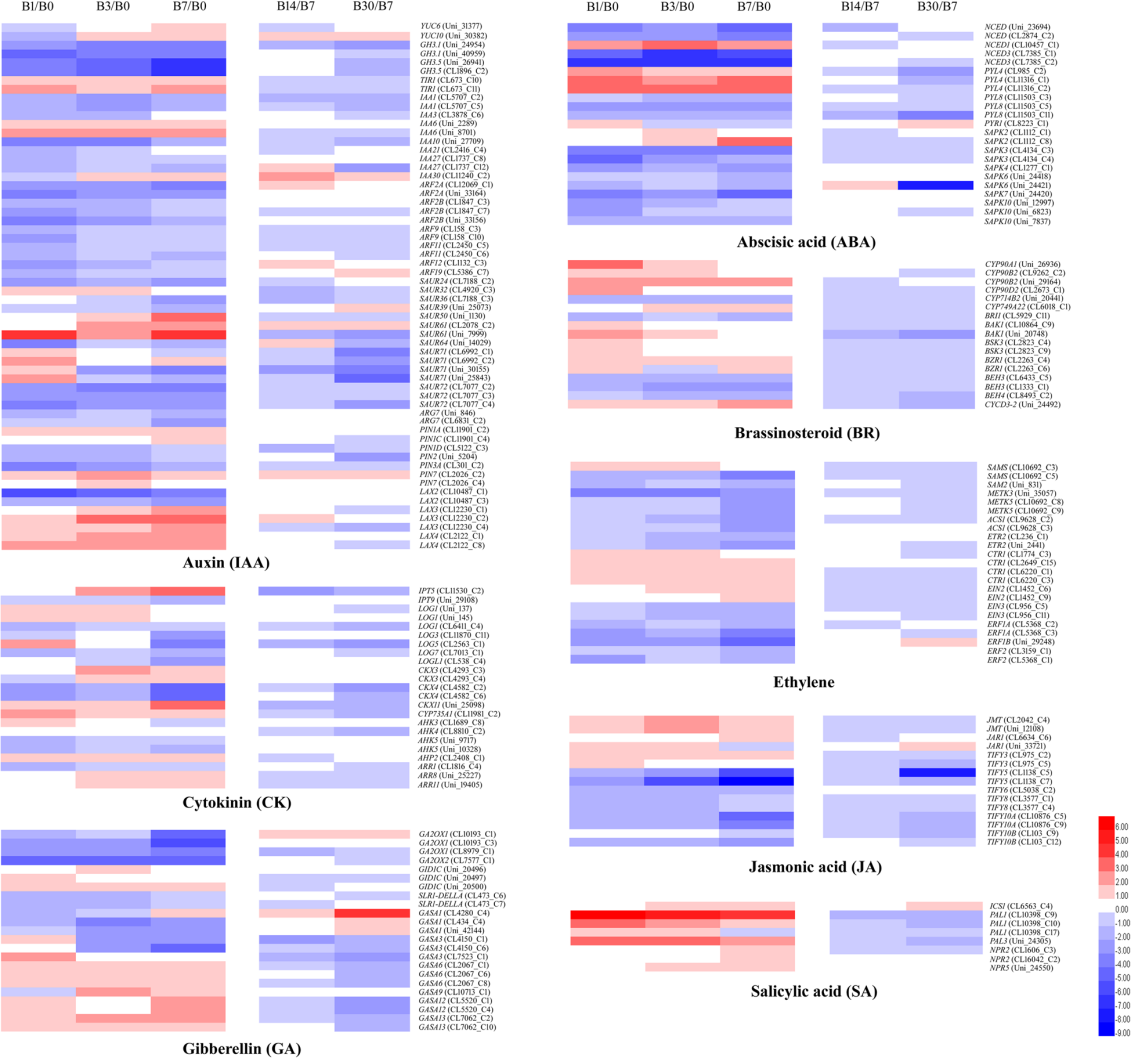
Expression patterns of DEGs related to hormone biosynthesis and signal transduction. Details are as described in the legend of Fig. 4

We identified numerous DEGs related to the synthesis and signal transduction of hormones, including IAA, CK, GA, ABA, BR, jasmonic acid (JA), and ethylene, during bulblet initiation and development. Notably, most of these DEGs were related to IAA (Fig. 7, Additional file 4: Table S4).

#### IAA

We identified 60 DEGs involved in auxin biosynthesis and signal transduction. As for the auxin synthesis pathway, two *YUCCA* genes were upregulated. Specifically, *YUC10* (Uni_30382) was downregulated on 1 DAT, but was significantly upregulated thereafter (Fig. 7). *GH3* genes (and especially, *GH3.1* (Uni_24954)), encoding IAA-amido synthetase, which conjugates IAA to amino acids, thus reducing the IAA concentration [25], was downregulated throughout bulblet formation.

Auxin signaling involves the activation or repression of gene expression by a class of auxin receptors, including the F-box protein TRANSPORT INHIBITOR RESPONSE1 (TIR1) and auxin response factor (ARF) [26, 27]. ARF activity is regulated in part through interactions with the auxin/indoleacetic acid (Aux/IAA) repressors [28], which are auxin-inducible and function as key regulators of auxin responses [29]. In our study, two *TIR1* genes (CL673_C10 and C11), *IAA6* (Uni_2289, Uni_8701), and, especially, *IAA30* (CL11240_C2) were upregulated during bulblet initiation (Fig. 7). IAA30 was also upregulated during bulblet development (Fig. 7). However, except for *ARF2A* (CL12069_C1), *ARF12* (CL1132_C3), and *ARF19* (CL5386_C7), *ARF* genes were downregulated or not significantly changed throughout bulblet formation. In several other species, ARF expression is up- or downregulated by auxin [30, 31], probably via feedback loops [32].

*SAUR* (small auxin-up RNAs) genes were previously reported to exhibit a rapid and specific increase in expression in response to exogenous auxin, which were capable to modulate auxin synthesis and transport and affect cell expansion [33]. In our study, several *SAUR* genes, especially, *SAUR50* (Uni_1130), *SAUR61* (CL2078_C2), and *SAUR61* (Uni_7999), were upregulated (Fig. 7), which may be explained by the increase in IAA content during bulblet formation.

Genes involved in the control of auxin distribution are also induced by auxin treatment. The *PIN-FORMED* (*PIN*) gene family of auxin efflux carriers and the like auxin1 (LAX) family of auxin influx carriers control auxin distribution to establish and maintain auxin concentration gradients in various plant tissues [28, 34]. Our results showed that *PIN* genes, including *PIN1A* (CL11901_C2), *PIN1C* (CL11901_C4), and *PIN7C* (L2026_C2), and *LAX* genes, except for *LAX2*, were significantly upregulated during bulblet initiation (Fig. 7).

#### ZR

In bud activity control in apical dominance, cytokinins antagonize auxin, and thus represent a possible second messenger for auxin signaling [35]. Consistent herewith, ZR contents showed opposite trends compared to IAA contents during bulblet initiation and development (Fig. 6b). However, genes related to CK biosynthesis, including *IPT5* (encoding adenosine phosphate-isopentenyltransferase, a key enzyme in CK biosynthesis), *LOG1* and *LOG5* (encoding cytokinin riboside 5′-monophosphate phosphoribohydrolase, a CK-activating enzyme that catalyzes the final step of bioactive CK synthesis), and *CYP735A1* (encoding a cytokine hydrolase that catalyzes the biosynthesis of trans-zeatin) (Fig. 7), were mostly upregulated at the early stage of bulblet formation. This may imply CK synthesis was enhanced during this stage. However, ZR contents were decreased (Fig. 6b), which may be due to an increase in CK degradation. Genes encoding cytokinin dehydrogenase (CKX), including *CKX3* (CL4293_C3 and C4) and *CKX11* (Uni_25098), were significantly upregulated during bulblet initiation (Fig. 7). Genes encoding histidine kinase (AHK), which is related to CK signal transduction, including *AHK3* to *AHK5*, were downregulated (Fig. 7), indicating a decrease in CK activity during bulblet formation.

#### GA

As for GA metabolism, four *GA2OX* genes encoding GA2-oxidase, which catalyzes the deactivation of bioactive GA or its precursors [36], were all significantly downregulated (Fig. 7), which may have contributed to the increase in endogenous GA content. As for GA signal transduction, three *GID1C* genes encoding a GA receptor and two *SLR1* genes encoding DELLA protein showed opposite expression patterns; they were up- and downregulated, respectively (Fig. 7).

Members of the plant-specific GA-stimulated Arabidopsis (*GASA*) gene family play roles in hormone response, defense, and development [37]. We identified numerous *GASA* genes that were differentially expressed during bulblet initiation and development, most of which were upregulated (Fig. 7). This implies that *GASA* genes may play important roles in regulating bulblet formation.

#### Aba

NCED, encoded by the *NCED* gene family, is a rate-limiting enzyme in ABA biosynthesis in plants. In our study, NCED genes, and, especially, NCED3 (CL7385_C1 and C2), were significantly downregulated (Fig. 7). In the ABA signal transduction pathway, ABA receptor *PYL8* genes were downregulated, whereas *PYL4* genes were upregulated. In addition, serine/threonine-protein kinase (*SAPK*) genes, which are involved in ABA signaling, were mostly downregulated (Fig. 7), implying that ABA signaling is suppressed.

#### Other hormones

We did not evaluate changes in endogenous BR, ethylene, JA, and SA contents, but we predicted their functions in controlling bulblet formation by analyzing changes in the expression levels of genes related to these hormones.

As for BR, *CYP* genes, encoding cytochrome P450 enzymes, which are involved in BR biosynthesis, were significantly upregulated (Fig. 7). This implied that BR may positively regulate bulblet formation. Consistent herewith, genes involved in BR signaling, including *BAK1* (BRASSINOSTEROID INSENSITIVE 1-ASSOCIATED KINASE 1), *BSK3* (BR signaling kinase 3), and *BZR1* (BRASSINAZOLE RESISTANT 1), were all upregulated. *CYCD3–2* (Uni_24492), which functions downstream of BR signaling, was also upregulated (Fig. 7).

The expression patterns of genes related to ethylene biosynthesis and signal transduction showed opposite trends to those of BR-related genes. In the ethylene biosynthesis pathway, *SAMS/METK* (encoding S-adenosylmethionine synthase) and *ACS1* (encoding 1-aminocyclopropane-1-carboxylic acid synthase) were significantly downregulated (Fig. 7). Ethylene signaling genes, including *ETR2* (encoding ethylene receptor 2), *EIN3* (encoding ETHYLENE INSENSITIVE 3), and *ERF* (encoding ethylene-responsive transcription factor 2), were significantly downregulated (Fig. 7). In addition, *CTR1* (encoding constitutive triple response 1, a negative regulator of ethylene signaling) was upregulated. Together, these results suggested that ethylene may negatively regulate bulblet formation.

*JMT* (encoding JA O-methyltransferase, which is related to JA biosynthesis) and *JAR1* (encoding JA-amido synthetase, which is related to JA signaling) were both upregulated (Fig. 7). However, the *TIFY* gene family, which includes *JAZ* genes (encoding JA ZIM domain-containing protein), the products of which act as repressors in JA signaling [38, 39], were mostly downregulated (Fig. 7). *ICS1* (encoding isochorismate synthase 1) and *PAL* (encoding phenylalanine a8mmonia-lyase), related to SA biosynthesis, and *NPR* genes, involved in SA-mediated signaling, were all significantly upregulated (Fig. 7). These results implied that JA and SA are both positive regulators of bulblet formation in *L. radiata*.

**Fig. 8:**
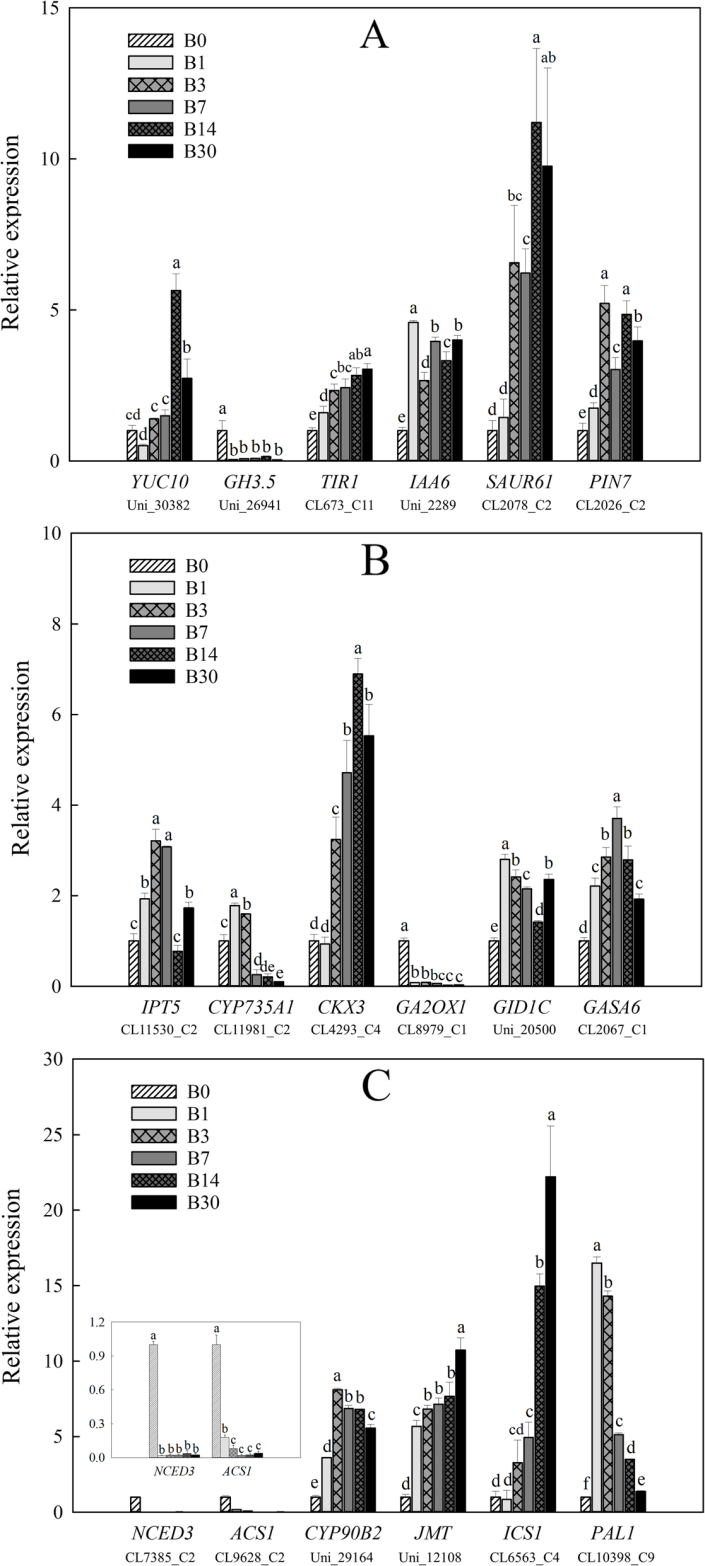
Changes in the expression levels of several genes involved in hormone biosynthesis and signal transduction during bulblet initiation and development. **a**: Genes related to auxin; **b**: genes related to CK and GA; **c**: genes related to ABA, ethylene, BR, JA, and SA. Details are as described in the legend of Fig. 5

We selected several genes involved in hormone biosynthesis and signal transduction that showed obvious and significant changes in gene expression in the RNA-seq analysis, for verification by RT-PCR (Fig. 8). In general, the results were consistent with the data obtained by RNA-seq, indicating that the RNA-seq data are reliable.

## Discussion

*L. radiata* is a perfect species to study the mechanism of bulblet initiation and development; unlike in other *Lycoris* species or other flowering bulbs (e.g., *Lilium* and *Hippeastrum*), in which bulblets appear randomly, the bulblets of *L. radiata* are preferentially produced on junctions of the innermost layer of scales and the basal plate, presenting initially as axillary buds and gradually developing into bulblets, which can be easily tracked (Fig. 1). Because of extensive preliminary observation, we could precisely collect tissue samples before axillary buds actually appeared. In the present study, we divided bulblets according to initiation and developmental stages based on the statuses of current axillary buds or bulblets (Fig. 1), and we evaluated changes in carbohydrate metabolism and hormone regulation during these stages.

Starch accumulation is vital for bulblet development in *Lycoris.* When newly formed bulblets gradually developed, starch contents in the bulblets increased (Fig. 3a). Soluble sugar contents also increased in the bulblets, but at earlier time points than starch (Fig. 3b). In previous studies on *Lycoris*, *Lilium*, and *Hippeastrum*, soluble sugars in the bulblets were found to be derived from starch degradation in the mother scales [6–8]. Consistent herewith, we observed a quick decrease in starch content in the mother scales, with differences among the three layers of scales (Fig. 3c). The outer scales showed the fastest decreases in starch and soluble sugar contents, followed by the middle scales (Fig. 3c, d), implying that starch is degraded mostly in the outer scales, from where the sugars are transported to the sites where bulblets will be initiated and grow.

In lilies and other bulbous ornamentals, sucrose is the main form of soluble sugars in bulbs [40], accounting for more than 70% of the soluble sugar fraction. Reducing sugars are largely converted into sucrose in the bulblet scales, and sucrose is used as a direct substrate for starch synthesis [8]. Sucrose metabolism is controlled by a series of enzymes, as shown in Fig. 4a. SUS is generally considered to be involved in sucrose hydrolysis [41], and *SUS* expression levels in this study were significantly upregulated during bulblet initiation (Figs. 4b, 5b), indicating that sucrose transported from the mother scales is used for the synthesis of starch, which would be beneficial for bulblet formation and enlargement. During stolon development into a new bulb in *Tulipa edulis*, starch is the predominant storage substance in new bulbs, and starch is synthesized by three major starch synthesis-related enzymes, i.e., AGPase, SS, and GBSS, from the product of sucrose cleavage catalyzed by SUS [42]. Consistent herewith, we found that AGPase, SS and GBSS activities increased throughout bulblet development *in L. radiata* (Fig. 5a). However, only AGPase showed a significant increase in activity during bulblet initiation (Fig. 5a). Gene expression patterns of these three enzymes were consistent with the changes in their activities (Figs. 4b, 5b), implying that AGPase and the genes encoding its subunits are major factors in promoting bulblet formation and development. AGPase is a major starch synthesis enzyme and has been demonstrated to play major roles in regulating bulb enlargement in *L. radiata* [24]. In support of our findings, in *Gladiolus hybridus*, the large subunit of AGPase, encoded by *GhAGPL1*, contributes to the quality and quantity of gladiolus corms and cormels, as silencing of *GhAGPL1* led to smaller corms and fewer cormels [43].

Bulblet formation is quite similar to axillary bud outgrowth, which is controlled by several hormones (especially, auxin and cytokinin) in model plants. The auxin transport canalization hypothesis suggested that polar auxin transport in the stem is required for bud outgrowth [44]. In our study, endogenous auxin content and gene expression related to auxin biosynthesis, signal transduction, and transport were significantly increased during bulblet initiation and development (Figs. 6a, 7, 8a), which is consistent with the action of auxin in controlling bud outgrowth in model plants. According to the canalization-based auxin transport model, the establishment of auxin transport channels in the stem is necessary for bud outgrowth, and the buds that develop first can inhibit auxin transport from those that would develop later, thus inhibiting their outgrowth [45, 46], this phenomenon is termed “apical dominance.” Similarly, bulblets firstly developed in certain zones may build a dominant channel of auxin transport, which could inhibit the emergence of bulblets on other scales.

CK has been proven to promote bud outgrowth [47]. In contrast herewith, in our study, ZR contents decreased during bulblet initiation (Fig. 6b). In bud outgrowth regulation, auxin has been shown to downregulate cytokinin synthesis at the node in the main stem [47]. Although CK triggers bud activation, once the bud grows out, auxin transported from the newly growing apex will inhibit CK synthesis [18]. In our study, gene expression related to CK biosynthesis first increased and then significantly decreased (Figs. 7, 8b), which may be related to the increase in auxin content in the growing bulblet. Meanwhile, CK degradation genes were upregulated throughout bulblet formation (Figs. 7, 8b), which may explain the decrease in endogenous CK.

GA contents increased during bulblet initiation and development (Fig. 6b), which was in agreement with the expression patterns of genes involved in GA synthesis and signal transduction (Figs. 7, 8b). Similar to auxin, GA inhibits bud outgrowth. In rice, the GA biosynthesis mutant *sd1* exhibits dwarfism, whereas the activation of GA signaling causes higher stature [18], and exogenous GA application significantly inhibited rice tiller bud outgrowth in our previous study [48]. However, GA reportedly stimulates axillary bud development in rose [49] and *Jatropha curcas* [50]. In addition, GA in the apical vasculature is necessary for normal plant development through its effect on auxin transport [51], and GA regulates PIN-FORMED abundance and is required for auxin transport-dependent growth and development in *Arabidopsis thaliana* [52]. Based on these results, we suggest that an increase in GA promotes bulblet outgrowth, which is also contributed to PIN-dependent auxin transport canalization in the vasculature.

ABA reportedly suppresses bud outgrowth [53], and is positively regulated by *BRC1* herein [54]. *BRC1* is specifically expressed in axillary buds and encodes a TCP transcription factor that is required to inhibit bud outgrowth and suppress branching [18]. Consistent herewith, both the ABA content and gene expression related to ABA biosynthesis and signaling were decreased during bulblet formation in *L. radiata* (Figs. 6b, 7, 8b), which implies an inhibitory effect of ABA on bulblet outgrowth.

As for other hormones including BR, ethylene, JA and SA, we indirectly investigated their involvement in bulblet formation by analyzing the expression of genes related to their synthesis and signaling (Figs. 7 and 8c). Based on our findings, we proposed a hypothesis that BR, JA, and SA promote, whereas ethylene inhibits bulblet formation. BR positively regulates rice bud outgrowth, as a BR biosynthesis mutant showed a reduction in tiller number [55], and overexpression of BR biosynthesis genes resulted in more branches [56]. While few studies have reported on the roles of JA, SA, and ethylene on bud outgrowth control in model plants, some studies have reported positive effects of JA and, especially, SA on rhizome or bulb development [57–59], which supports our above-mentioned hypothesis.

**Fig. 9:**
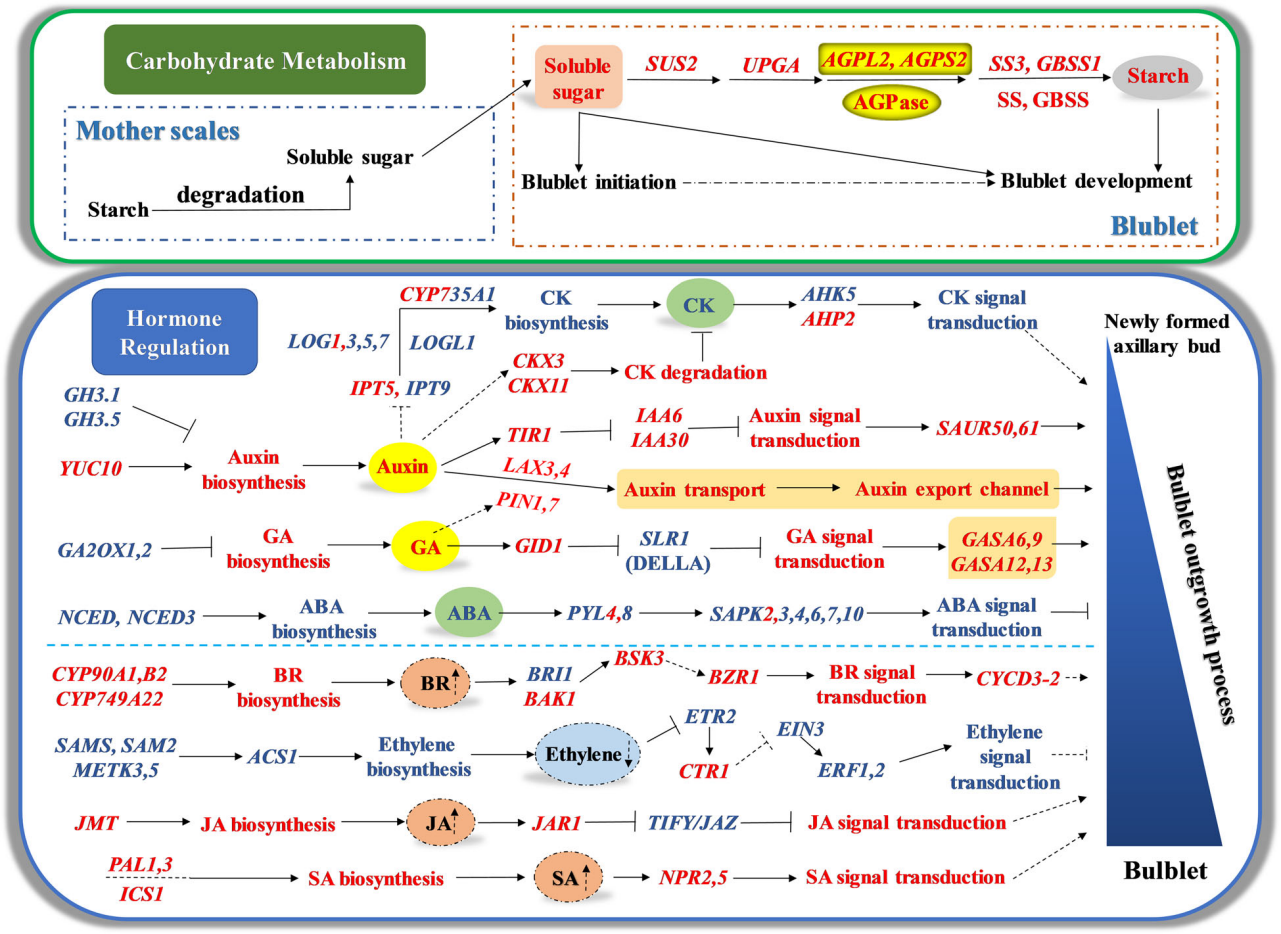
A proposed model to explain carbohydrate metabolism and endogenous hormone regulation during bulblet initiation and development in *L. radiata*

Based on our major findings, we propose a model to explain carbohydrate metabolism and endogenous hormone regulation during bulblet initiation and development in *L. radiata* (Fig. 9). In this model, soluble sugars, mainly derived from starch degradation in the outer scales, promote bulblet initiation and are used to synthesize starch in the bulblet for further development. This latter process is under the control of several genes involved in carbohydrate metabolism, especially, genes encoding AGPase, a crucial starch synthesis enzyme. Based on our results, we suggested that enhancing sugar content could improve the proliferation efficiency of bulblets, which could be realized through the application of sugars (e.g., sucrose) in the growth medium of bulb sections in tissue culture production of *L. radiata* and has been verified in the bulblet regeneration of lily in vitro [60]. Also, we proposed that overexpression of genes encoding AGPase subunits through transgenic and other molecular biology approaches could lead to the significant promotion of AGPase activities in *L. radiata* bulbs, which may result in a higher proliferation efficiency of bulblets, and this suggestion has been preliminarily verified in gladiolus [43].

In addition, we also proposed a model to explain the relationship and interaction between changes in endogenous hormones and expression of genes involved in hormone biosynthesis and signal transduction during bulblet initiation and development (Fig. 9). The bulblet outgrowth process needs the continuous support of endogenous IAA and GA, as their content kept increasing, which may be regulated by the up-regulation of IAA synthesis gene *YUC10*, and the downregulation of IAA and GA degradation genes *GH3.1*, *GH3.5,* and *GA2OX1–2,* respectively. The effect of IAA and GA in promoting bulblet development (e.g., cell elongation and expansion) may be controlled by *SAUR* and *GASA* genes, which were under the downstream regulation by their signal transduction genes, including *TIR1*, *IAA* genes and *GID1*, *SLR1*(DELLA protein) respectively. Moreover, auxin transport from the newly formed bulblets benefits their subsequent outgrowth, which may be regulated by the up-regulation of auxin transport genes *LAX3,4* and *PIN1,7*. Endogenous ZR and ABA content showed similar decrease during bulblet growth, which may be regulated by the down-regulation of their synthesis genes *IPT5*, *LOG3,5,7* and *NCED*, *NCED3,* respectively, and resulted in the downregulation of signal transduction genes of CK and ABA. However, expression levels of *IPT9*, *LOG1* and *CYP735A1* showed an increase during bulblet initiation, which implies an accelerated CK biosynthesis; thus, we proposed that the decrease in ZR content was mainly caused by the upregulation of CK degradation genes *CKX3* and *CKX11*. The decrease and increase in the expression levels of CK synthesis and degradation genes respectively may partly be regulated by the increasing auxin content, which should be investigated in future. In terms of BR, ethylene, JA, and SA, we did not determine the changes in their content directly, but we found that expression levels of BR, JA, SA, and ethylene biosynthesis genes showed an increase and decrease, respectively, implying that BR, JA and SA content were increased during bulblet development, while ethylene content was decreased. In addition, expression patterns of genes that were positively or negatively regulated in signal transduction were consistent with those of their synthesis genes, supporting our hypothesis about changes in BR, JA, SA, and ethylene content. Based on these results, we preliminarily assessed the effect of endogenous hormones on bulblet initiation and development in *L. radiata*, which could provide theoretical basis for the application of exogenous hormones to improve proliferation efficiency of bulblet in production.

## Conclusions

In this study, we analyzed the changes in the content of carbohydrates and endogenous hormones as well as the gene expression patterns during bulblet initiation and development in *L. radiata*. Based on these results, we proposed that soluble sugars derived from the mother scales were transported to the zones where bulblet was initiated, supporting the subsequent development of bulblets. When bulblet gradually developed, starch content increased, which was mainly regulated by genes encoding AGPase, a crucial enzyme associated with starch synthesis. In addition, we found that endogenous IAA and GA content showed an increase, while ZR and ABA content decreased during bulblet initiation and development, which were consistent with the expression patterns of genes involved in IAA, GA_3_, and ABA synthesis and signal transduction, implying that increasing IAA and GA_3_ content were beneficial for bulblet outgrowth, while ABA was not. We also hypothesized that the decrease in ZR content may be regulated by the inhibition of CK synthetic genes through an increase in auxin and promotion of CK degradation, which needs to be verified in future studies. Furthermore, the expression levels of BR, JA, and SA biosynthesis genes and positively regulated signal transduction genes were increased, although this trend was the opposite in the case of ethylene, indicating that BR, JA, and SA have promotive, while ethylene has suppressive effects, respectively, on bulblet formation.

## Methods

### Plant materials and treatment

*L. radiata* materials used in this study were originally obtained from the *Lycoris* germplasm repository of Nanjing Botanical Garden Mem. Sun Yat-Sen (NBG) by YCZ and QZL under legal permission on May 20, 2016, and a voucher specimen (samples NO. NAS00571237) of this material has already been deposited at the herbarium of NBG. These bulbs were transplanted into the experimental base at the Shanghai Academy of Agricultural Sciences (Qingpu District, Shanghai, China) for more than 2 years. When *L. radiata* plants were dormant and without leaves at the end of July, bulbs with a diameter of 2.7 ± 0.2 cm were collected. After removal of the roots and dried scales and surface sterilization, the bulbs were chipped into four sections on average and placed on enamel trays, as described previously [6]. The sections were covered with a gauze that was sprayed with distilled water twice daily to keep it moist. After these pre-treatments, the sections were placed in plant growth chambers for bulblet formation, under a 14-h light/10-h dark photoperiod (6000 lx), at 25/20 °C (day/night), and with a relative humidity of 80%. The experiment was started on July 31, 2018, and lasted 2 months. Two hundred bulb sections were prepared in the experiment, including three replicates, and each replicate consisted of 66 or 67 sections.

### Sampling

We previously observed that bulblets appear and develop at junctions of the innermost layer of scales and the basal plate, where axillary buds are formed that gradually develop. Bulb sections were collected on days 0, 1, 3, 7 DAT, and samples of tissues surrounding the zones of axillary bud emergence were collected. After the axillary buds grew out, the newly formed bulblets of sections were collected weekly during the two-month experiment. Our previous study revealed that the scales of *L. radiata* bulbs can be separated into three layers based on a morphological index, and each layer may have different roles in regulating bulb development [24]. Thus, the three layers of each section were also separately sampled. All the materials were frozen in liquid nitrogen for 30 min and stored at − 70 °C.

### Starch and soluble sugar content measurements

The contents of starch and total soluble sugars were measured by traditional anthrone colorimetry [42]. Samples were ground in liquid nitrogen, and approximately 0.5 g of powder was incubated with 4 ml of 80% ethanol at 80 °C for 30 min. Then, the extraction solution was centrifuged at 8000×*g* for 20 min. After decolorization with activated carbon, soluble sugars in the supernatant were measured. The precipitates were successively suspended in 9.2 M and 4.6 M HClO_4_ to extract the starch after removing the ethanol-soluble sugar residues. Total soluble sugar and starch concentrations were then determined using the anthrone reaction.

### Starch synthesis enzyme activity measurements

Enzyme extraction and determination of the activities of starch synthesis enzymes (AGPase, SS, and GBSS) were carried out as previously reported [61, 62]. Thoroughly mixed frozen bulb tissues (0.5 g) were prepared for each sample. All procedures were conducted at a temperature of 0 °C to 4 °C. Samples were ground as described above and.

extracted with buffer solution [5 ml g^− 1^ sample fresh weight (FW)] containing 100 mM 4-(2-hydroxyethyl)-1-piperazineethanesulfonic acid (HEPES)-NaOH frozen extraction buffer (pH 7.5), 8 mM MgCl_2_, 2 mM ethylenediaminetetraacetic acid (EDTA), 50 mM 2-mercaptoethanol, 12.5% glycerol, and 1% (0.01 g/ml) insoluble.

polyvinylpyrrolidone-40). The homogenate was then centrifuged at 30000×*g* for 30 min, and the supernatant was used for the determination of AGPase and SSS, while the sediment was used for GBSS. The activities of AGPase, SSS, and GBSS were measured following the method described previously [61, 62]. The enzymes were compared based on soluble protein content, which was determined by a modified Bradford method [63]. All treatment experiments consisted of three independent replicates.

### Endogenous plant hormone measurements

The levels of IAA, ZR, GA_3_, and ABA were determined at Qingdao Sci-tech Innovation Quality Testing Co., Ltd. (Qingdao, China). Sample extraction and purification were carried out as described previously [64], with a modification. Approximately 0.2 g of sample was first ground in liquid nitrogen. After adding 1 ml of cold 50% acetonitrile (v/v) at 4 °C, the samples were further ground in a vibration mill at 50 Hz for 2 min, and then ultrasound-extracted for 3 min. After incubation at 4 °C for 4 h, the samples were centrifuged at 12,000×*g* at 4 °C for 10 min. The supernatant was purified using an Oasis HLB purification column (Waters) and was collected in a plastic microtube. The samples were dried under N_2_, dissolved in 200 μl of 30% acetonitrile (v/v), and filtered using 0.22-μm membrane filters.

The purified product was subjected to high-performance liquid chromatography-tandem mass spectrometry (TSQ Quantum Ultra, Thermo) analysis, using a C18 (Agilent Technologies) column (2.1 mm × 100 mm, 1.8 μm) at a flow rate of 0.3 ml min^− 1^, with the gradients of solvent A (0.1% methanoic acid) and B (acetonitrile) set according to the following profile: 1 min, 95% A + 5% B; 15 min, 20% A + 80% B; 16 min, 100%B; 19 min, 95% A + 5% B. The column temperature was set at 40 °C and the injection volume was 5 μl. MS conditions were as follows: the spray voltage was 3500 V (ESI –) and 4000 V (ESI +) respectively, and the atomizing temperature was 330 °C.

The external standard method was used to determine the hormone contents. Calibration curves for IAA, ZR, GA_3_ and ABA standards were obtained using seven or eight concentrations (0, 1, 5, 10, 50, 100, 500, and 1000 ng/ml) (Additional file 5: Fig. S1). TIC chromatograms of standards are shown in Additional file 6: Fig. S2.

### RNA extraction

Total RNA extraction and cDNA synthesis were carried out as described previously [65]. Total RNA was extracted according using an RNAprep Pure Plant Kit (Tiangen Biotech, Beijing, China) per the manufacturer’s instructions. After measuring the RNA quantity and quality using a NanoDrop 2000 Spectrophotometer (Thermo Fisher Scientific, USA), we selected those samples with A_260/280_ = 1.8–2.2 for library preparation. Three biological replicates were used for RNA-Seq.

### cDNA library construction and sequencing

The cDNA library was constructed using an mRNA-Seq Sample Preparation Kit (MGIEasy™ mRNA Library Prep Kit, MGI, Shenzhen, China), according to the manufacturer’s instructions; as described previously [66]. Poly(A) mRNA was enriched by oligo magnetic adsorption. The enriched mRNA was fragmented and reverse-transcribed into double-stranded cDNAs with an N6 random primer. Sequencing adaptors were linked to the purified cDNA, and 15 double-strand libraries were generated by PCR amplification. The libraries were sequenced on a BGISEQ-500 platform at the Beijing Genomics Institute (www.genomics.org.cn, Shenzhen, China).

### *De-novo* transcriptome assembly

Low-quality sequences, including sequences with ambiguous bases, low-quality reads, and reads with adaptors, were removed from the paired-end raw reads. Only clean reads were used in subsequent analyses. The high-quality reads were assembled using Trinity with default parameters to construct unique consensus sequences [67, 68].

### Analysis of differential gene expression

Unigene expression levels were calculated based on FPKM values. Then, DEGs among the sample groups were identified using the NOISeq package [69]. DEGs were identified based on a false discovery rate < 0.05 and |log_2_ foldchange| ≥1.

### Functional annotation of unigenes

Unigenes were annotated by BLASTx against seven public databases, including Nr, Nt, SwissProt, KOG, Pfam, GO, and KEGG. GO annotation was performed using the Blast2GO software, as described previously [70].

### qRT-PCR assays

Approximately 500 ng of total RNA was used to prepare first-strand cDNA using the PrimeScript RT Reagent Kit (TaKaRa, Dalian, China) per the manufacturer’s instruction. The cDNA was used for qRT-PCR, which was carried out on an ABI 7500 Fast sequencer using SYBR Premix Ex Taq™ (Takara, Kyoto, Japan), as described previously [15]. *Actin7* (Uni_17610) was used as a reference gene. Three biological replicates were included per treatment. Primers used are listed in Additional file 7: Table S5.

### Statistical analyses

All statistical analyses were conducted using SPSS 16.0. Means of values were compared by standard analysis of variance followed by least significant difference tests, *P* < 0.05 was considered significant.

## Supplementary information

**Additional file 1: Table S1.** Mapping results of RNA-seq reads.

**Additional file 2: Table S2.** The quality of assembled unigenes.

**Additional file 3: Table S3.** The information of differentially expressed unigenes involved in carbohydrate metabolism.

**Additional file 4: Table S4.** The information of differentially expressed unigenes involved in hormone biosynthesis and signal transduction.

**Additional file 5: Figure S1.** Calibration curves for IAA, ZR, GA3 and ABA standards obtained from HPLC-MS/MS analysis. a: IAA; b: ZR; c: GA_3_; d: ABA.

**Additional file 6: Figure S2.** TIC chromatograms of standards (1 μg/ml) obtained from HPLC-MS/MS analysis. a: ZR, IAA and ABA standards at ESI + mode; b: GA_3_ standard at ESI – mode.

**Additional file 7: Table S5.** Primers used in qRT-PCR.

## Data Availability

All relevant supplementary data is provided within this manuscript as additional files 1, 2, 3, 4, 5, 6 and 7. All clean read data produced in this study have been deposited in the NCBI Sequence Read Archive (http://www.ncbi.nlm.nih.gov/sra/) under the accession numbers SRR10198454, SRR10198463 and SRR10198464 (B0), SRR10198451–SRR10198453 (B1), SRR10198448–SRR10198450 (B3), SRR10198447, SRR10198461, and SRR10198462 (B7), SRR10198458– SRR10198460 (B14), and SRR10198455–SRR10198457 (B30).

## Abbreviations

ABA: Abscisic Acid
ACN: Acetonitrile
ACS1: 1-aminocyclopropane-1-carboxylic acid synthase
AGP: Glucose-1-phosphate adenylyltransferase
AMY: Alpha-amylase
ARF: Auxin response factor
Aux/IAA: Auxin/indoleacetic acid
BAK1: BRASSINOSTEROID INSENSITIVE 1-ASSOCIATED KINASE 1
BMY: Beta amylase
BR: Brassinosteroid
BRC1: BRANCHED1
BSK3: BR signaling kinase 3
BZR1: BRASSINAZOLE RESISTANT 1
CK: Cytokinin
CKX: Cytokinin dehydrogenase
CTR1: Constitutive triple response 1
DAT: Days after treatment
DEGs: Differentially expressed genes
ETR2: Ethylene receptor 2
EIN3: ETHYLENE INSENSITIVE 3
ERF: Ethylene-responsive transcription factor 2
FPKM: The fragments per kilobase of transcript sequence per million base pairs sequenced
FRK: Fructokinase
GA: Gibberellin
GASA: GA-stimulated Arabidopsis
GBSS: Granule-bound starch synthase
GO: Gene Ontology
HPLC-MS/MS: High-performance liquid chromatography-tandem mass spectrometry
IAA: Indole-3-acetic acid
ICS1: Isochorismate synthase 1
INV: Invertase
ISA: Isoamylase
JA: Jasmonic Acid
JAR1: JA-amido synthetase
JAZ: JA ZIM domain-containing protein
JMT: JA O-methyltransferase
KEEG: Kyoto Encyclopedia of Genes and Genomes
KOG: Clusters of Orthologous Groups of proteins
LAX: Like auxin1
NCBI: National Center for Biotechnology Information
NCED: 9-cis-epoxycarotenoid dioxygenase
Nr: NCBI non-redundant
Nt: Nucleotide
PAL: Phenylalanine ammonia-lyase
PGM: Phosphoglucomutase
PIN: PIN-FORMED
RNA-Seq: RNA sequencing
RT-PCR: Reverse transcription quantitative PCR
SA: Salicylic acid
SAMS: S-adenosylmethionine synthase
SAPK: Serine/threonine-protein kinase
SAUR: Small auxin-up RNAs
SBE: Starch branching enzyme
SSS: Soluble starch synthase
SUS: Sucrose synthase
TB1: TEOSINTE BRANCHED1
TIC: Total ion chromatogram
TIR1: TRANSPORT INHIBITOR RESPONSE1
UGP: UTP-glucose-1-phosphate uridylyltransferase
ZR: Zeatin riboside
