# Supplementary material for: Changes in carbohydrate metabolism and endogenous hormone regulation during bulblet initiation and development in Lycoris radiata
Source: BMC Plant Biol. 2020 Apr 25;20:180. doi: 10.1186/s12870-020-02394-4 (PMC7183599; doi:10.1186/s12870-020-02394-4)
Supplement: Supplementary file 5 — Additional file 5: Figure S1. Calibration curves for IAA, ZR, GA3 and ABA standards obtained from HPLC-MS/MS analysis. a: IAA; b: ZR; c: GA3; d: ABA. [file 12870_2020_2394_MOESM5_ESM.pdf]

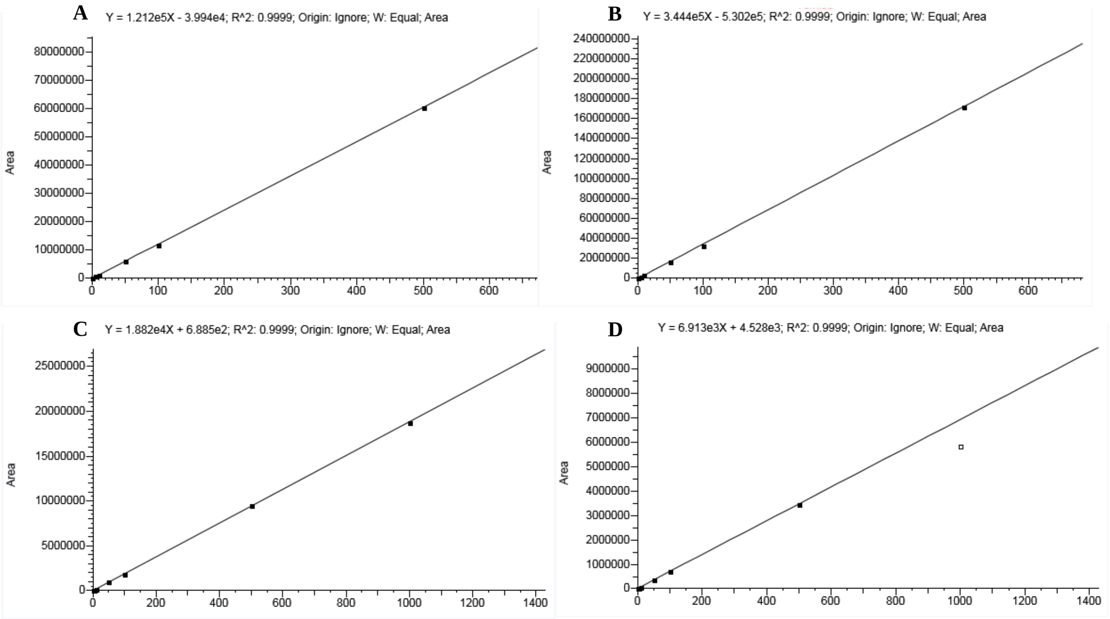

Fig. S1 Calibration curves for IAA, ZR, GA_3 and ABA standards obtained from HPLC-MS/MS analysis. a: IAA; b: ZR; c: GA_3; d: ABA.
