# Supplementary material for: Changes in carbohydrate metabolism and endogenous hormone regulation during bulblet initiation and development in Lycoris radiata
Source: BMC Plant Biol. 2020 Apr 25;20:180. doi: 10.1186/s12870-020-02394-4 (PMC7183599; doi:10.1186/s12870-020-02394-4)
Supplement: Supplementary file 6 — Additional file 6: Figure S2. TIC chromatograms of standards (1 μg/ml) obtained from HPLC-MS/MS analysis. a: ZR, IAA and ABA standards at ESI + mode; b: GA3 standard at ESI – mode. [file 12870_2020_2394_MOESM6_ESM.pdf]

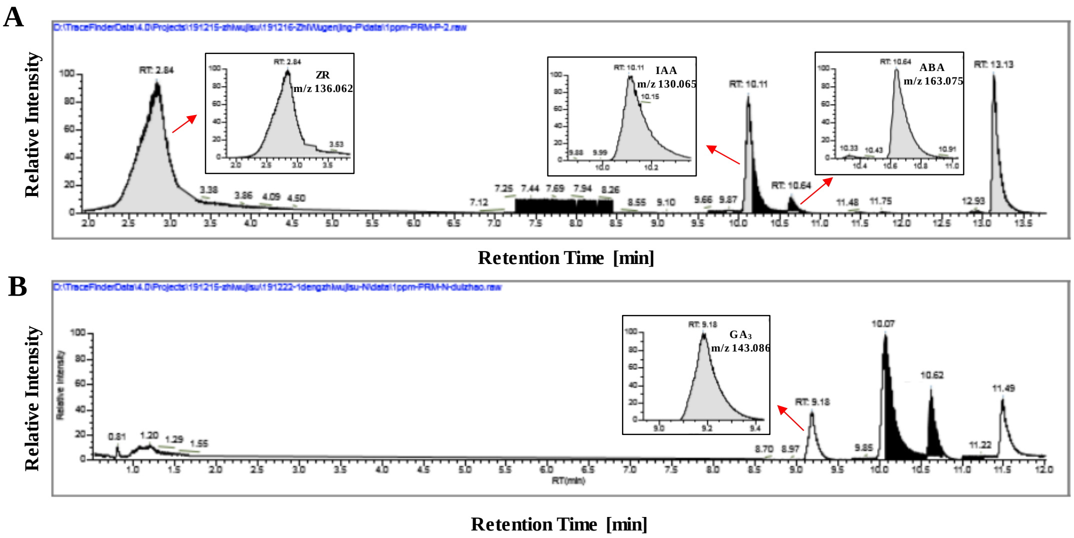

Fig. S2 TIC chromatograms of standards (1 μg/ml) obtained from HPLC-MS/MS analysis. a: ZR, IAA and ABA standards at ESI+ mode; b: GA_3 standard at ESI- mode.
